# 

**Published:** 1899-07-15

**Authors:** 


**Notices of Births, Deaths, and Marriages** are inserted in this column at a charge of 2s. 6d. for an announcement not exceeding four lines.

### NOTICE TO CORRESPONDENTS.

All MS., Letters, Books for Review, and other matters intended for the Editor should be addressed The Editor, "The Hospital," The Hospital Building, 28 & 29, Southampton Street, Strand, W.C.

The Editor cannot undertake to return rejected MS., even when accompanied by stamped directed envelope.

**Notice to Advertisers and Subscribers.**

All Advertisements, Orders for Copies of The Hospital, and Business Communications should be addressed to **THE MANAGER (Not to the Editor)**, The Hospital, The Hospital Building, 28 & 29, Southampton Street, Strand, London, W.C.

The Cost of Subscription, post free, is as follows:—Per week, 2½d.; per quarter, 2s. 8d.; per half-year, 5s. 3d.; per annum, 10s. 6d. Foreign:—Per annum, 15s. 2d., payable, in all cases, in advance.

**NOTICE.—Vol. XXV. of "The Hospital" (October, 1898, to March, 1899), in handsome cloth binding, is now on sale at the Office of this Journal, price 7s. 6d. Cases for binding the Numbers contained in this Volume 1s. 6d. Index to Vol. XXV., 2½d., post free.**

**The Monthly Part for July is now ready. Price 6d., by post 8d.**

### BOOKS RECEIVED.

Henry Kimpton.
"Gleet." By Gerald Dalton.

The Trustees of the Boston City Hospital.
"Medical and Surgical Reports."

John Murray.
"The Journal of the Royal Agricultural Society."

P. S. King and Son.
"Report of the Poor Law Conference."

J. Maxwell and Sons.
"Annual Report." By J. Maxwell Ross, M.A., M.B., Medical Officer for the County of Dumfries.

Cassell and Co.
"Intestinal Obstruction." By Frederick Treves, F.R.C.S. New and revised edition.

The Scientific Press.
"The Commonwealth of the Body." By G. A. Hawkins-Ambler, F.R.C.S.

The "China Mail" Office, Hong Kong.
"On the Decline of Scurvy Afloat." By Surgeon C. Marsh Beadwell, R.N.

London Anti-Vivisection Society.
"Pasteur, Hydrophobia, and Sero-therapy." By Dr. A. Lutaud.

**Periodicals and Pamphlets.** — St. Bartholomew's Hospital Journal, Sanitary Record, Leeds Hospital Magazine, Academy, Australasian Medical Gazette, Medical Press, Charity Organisation Review, Guy's Hospital Gazette, Medical and Surgical Review of Reviews.

